# Changing Management of Clinical Low-Stage Testicular Cancer

**DOI:** 10.1100/tsw.2005.97

**Published:** 2005-10-16

**Authors:** Timothy Gilligan

**Affiliations:** Department of Hematology and Medical Oncology, Cleveland Clinic Foundation, Cleveland, Ohio, USA

**Keywords:** testicular cancer, germ cell tumors, seminomas, nonseminomas, radiotherapy, chemotherapy

## Abstract

Stage I and II testicular germ cell tumors (GCTs) are almost always cured with appropriate treatment and most ongoing research regarding these tumors focuses on minimizing treatment toxicity. The management of clinical stage I testicular GCTs has grown more complicated due to the emergence of a brief course of chemotherapy as an additional treatment option for stage I seminomas and stage I nonseminomas. In addition, growing concern about radiation-induced cancers and other late toxicity has dulled enthusiasm for radiotherapy as a treatment for stage I seminomas. However, recent randomized trials have shown that radiotherapy doses and field sizes can be lowered without compromising cure rates and it is possible that this reduction in radiation exposure will reduce the rate of secondary cancers. At this point in history, stage I patients have three treatment options following radical orchiectomy: adjuvant (sometimes called “primary”) chemotherapy (carboplatin for seminomas and the combined regimen of bleomycin, etoposide, and cisplatin for nonseminomas), surveillance, and either retroperitoneal lymph node dissection (for nonseminomas) or radiotherapy (for pure seminomas). Clinical studies have made it possible to identify subgroups of patients at high and low risk for relapse and this has made it possible to tailor treatment decisions to the individual patient's postorchiectomy relapse risk.

## INTRODUCTION

Chemotherapy is playing a growing role in the management of clinical low-stage testicular cancer. Early stage testicular cancer has long been associated with a near-perfect, long-term, disease-specific survival. Clinical stage I pure seminomas are cured in 99.5% of patients while clinical stage I nonseminomas have a cure rate of about 99% (Tables 1 and 2). Early stage II disease (stage IIA) is associated with almost as favorable a prognosis, with roughly 95% of seminomas and nonseminomas alike cured. It is important to note that men with persistently elevated or rising serum tumor markers, but no radiographic evidence of metastatic disease, are generally treated with multiagent, cisplatin-based chemotherapy for disseminated disease unless an alternative source for the elevated tumor markers can be established[1,2].

*Author mailing address: Taussig Cancer Center, Cleveland Clinic Foundation, 9500 Euclid Avenue / R-35, Cleveland, OH 44195
©2005 with author.

**TABLE 1**
**Results of Carboplatin, Radiation, and Surveillance for Stage I Seminoma**

| Study | N | Median Follow-Up | Relapses | % Relapsed | 5-Year OS (%) | 5-Year DSS (%) | 5-Year RFS (%) |
|---|---|---|---|---|---|---|---|
| SURVEILLANCE | | | | | | | |
| Warde[a] [56] | 638 | 84 | 121 | 19.0 | 97.7 | 99.3 | 82.3 |
| Daugaard[80] | 394 | 60 | 69 | 17.5 | 98.6 | 100 | 83 |
| Totals | 1032 | | 190 | 18.4 | | | |
| CARBOPLATIN (2 cycles) | | | | | | | |
| Oliver[10] | 57 | 128 | 1 | 1.8 | 100.0 | 100 | 98.2 |
| Krege[7] | 43 | 28 | 0 | 0 | | 100[b] | 100[b] |
| Dieckmann[11] | 32 | 45 | 0 | 0 | 100.0 | 100 | 100 |
| Aparicio[9] | 60 | 52 | 2 | 3.3 | 96.7 | 100 | 96.6 |
| Steiner[15] | 108 | 60 | 2 | 1.9 | 100.0 | 100 | |
| Reiter[14] | 107 | 74 | 0 | 0 | 94.4 | 100 | 100 |
| Kratzik[59] | 39 | 20 | 1 | 2.6 | N/A | N/A | N/A |
| Aparicio[57] | 204 | 20 | 5 | 2.5 | 100.0[c] | 100[c] | 96.4[c] |
| Totals | 650 | | 11 | 1.7 | | | |
| RADIATION | | | | | | | |
| Warde[4] | 194 | 97 | 11 | 5.7 | 97.0 | 100.0 | 94.5 |
| Logue[54] | 431 | 62 | 15 | 3.5 | 98.0 | 99.8 | 96.3 |
| Bamberg[51] | 483 | 55 | 18 | 3.7 | | 99.8 | 95.8 |
| Bauman[99] | 169 | 90 | 5 | 3.0 | | 100.0 | 95.0 |
| Giacchetti[100] | 184 | 216 | 4 | 2.2 | 96.0 | 97.0 | |
| Fossa[42] | 478 | 54 | 18 | 3.8 | 99.6 | | 96.0 |
| Santoni[46] | 487 | 105 | 21 | 4.3 | 97[d] | | |
| Classen[52] | 675 | 61 | 26 | 3.9 | | 99.6 | 95.8 |
| Oliver[13] | 904 | 36 | 36 | 4.0 | 99.4[e] | 99.9[e] | 96.6[c] |
| Jones[28] | 625 | 61 | 21 | 3.4 | 99 | 99.8 | 96.7 |
| Totals | 4630 | | 175 | 3.8 | | | |

OS = overall survival, DSS = disease-specific survival, RFS = relapse-fee survival.

[a] Pooled international analysis.
[b] Results at 28 months median follow-up.
[c] 3-year survival.
[d] 10-year survival.
[e] Results at 36 months median follow-up.

With such favorable outcomes, attention has turned to minimizing short- and long-term complications of treatment without compromising treatment efficacy. The first step in this direction occurred when published studies reported that close surveillance for stage I testicular germ cell tumors (GCTs) produced indistinguishable, long-term, disease-specific, survival rates when compared to radiation therapy for stage I seminoma and retroperitoneal lymph node dissection (RPLND) for stage I nonseminomas[3,4,5,6].

**TABLE 2**
**Results of Chemotherapy, RPLND, and Surveillance for Stage I Testicular Nonseminomatous GCTs**

| Study | N | Median Follow-Up | Relapses | % Relapsed | OS (%) | DSS (%) | GCT-Related Deaths |
|---|---|---|---|---|---|---|---|
| SURVEILLANCE | | | | | | | |
| Freedman[75] | 259 | 30 | 70 | 27.0 | 98.0 | 98.0 | 1.2% |
| Read[76] | 396 | 60 | 100 | 25.3 | 98.0 | 99.0 | 1.3% |
| Sogani[77] | 105 | 135.6 | 27 | 25.7 | | 97.1 | 2.9% |
| Colls[78] | 248 | 53 | 70 | 28.2 | 97.6 | 98.0 | 1.2% |
| Spermon[79] | 90 | 92.4 | 23 | 25.6 | | 98.5 | 1.1% |
| Daugaard[80] | 301 | 60 | 86 | 28.6 | 98.6 | 100.0 | 0.0% |
| Gels[81] | 154 | 84 | 42 | 27.3 | 98.7 | 98.7 | 1.3% |
| Alexandre[82] | 88 | 51.6 | 24 | 27.3 | | 98.9 | 1.1% |
| Totals | 1641 | | 442 | | | | 1.1% |
| | | | | | | | |
| CHEMOTHERAPY | | | | | | | |
| Pont[25] | 29 | 79 | 2 | 6.9 | 93.0 | 96.5 | 3.45% |
| Cullen[17] | 114 | 48 | 2 | 1.8 | 98.0 | 98.0 | 1.75% |
| Bohlen[18] | 58 | 93 | 2 | 3.4 | 100.0 | 100.0 | 0.00% |
| Abratt[19] | 20 | 31 | 0 | 0.0 | N/A | 100.0 | 0.00% |
| Ondrus[23] | 18 | 36 | 0 | 0.0 | 100.0 | 100.0 | 0.00% |
| Mourey[20] | 64 | 51 | 1 | 1.6 | 100.0 | 100.0 | 0.00% |
| Oliver [21] | 148 | 33 | 6 | 4.1 | 97.3 | 97.3 | 1.35% |
| Amato[22] | 68 | 38 | 1 | 1.5 | 100.0 | 100.0 | 0.00% |
| Hendry[24] | 60 | 60 | 2 | 3.3 | 98.3 | 98.3 | 1.67% |
| Totals | 579 | | 16 | 2.8 | | | 1.04% |

| Author | N | N PSI | Median Follow-Up (Months) | % Receiving Adjuvant Chemotherapy for PS II | % Receiving Any Chemotherapy | Relapse Rate Among PSI | Relapse Rate Among CSI | OS | DSS | Deaths Related to GCT |
|---|---|---|---|---|---|---|---|---|---|---|
| Spermon[79] | 101 | 70 | 83 | 31 | 38 | 10.0% | 6.9% | 98% | 98% | 1.0% |
| Hermans[85] | 292 | 226 | 46 | 12 | 22 | 10.2% | 10.3% | NR | NR | NR |
| Klepp[84]* | 99 | 85 | 40 | 14 | 27 | 15.3% | 13.1% | 100.0% | 100 | 0% |
| Donohue[83] | 378 | 266 | 75 | 13 | 25 | 11.7% | 14.0% | 99.2% | 99.2% | 0.8% |
| Stephenson[2] | 108 | 72 | 30 | 16 | 20 | 3% | 4% | N/A | 100% | 0 |
| Totals | 978 | 719 | | 15 | 25 | 10.8% | 11.5% | | | 0.7% |

PSI = pathological stage I; CSI = clinical stage I.

*All pathological stage II patients in this series received adjuvant chemotherapy.

More recently, adjuvant chemotherapy (in other words, chemotherapy given as primary treatment following orchiectomy) as treatment for clinical stage I disease has produced similarly outstanding, disease-specific survival as surveillance, radiation therapy (for seminomas), and RPLND (for nonseminomas)[7,8,9,10,11,12,13,14,15,16,17,18,19,20,21,22,23,24,25]. More impressively, adjuvant chemotherapy has produced the lowest relapse rates of any treatment modality for seminomas and nonseminomas alike. As a result, chemotherapy is now accepted by many as an option for first-line therapy for stage I testicular GCTs[26,27]. For seminomas, chemotherapy consists of either one or two cycles of single-agent carboplatin and for nonseminomas, the regimen is two cycles of bleomycin, etoposide, and cisplatin (BEP). For those patients choosing radiation therapy for stage I seminomas, there are new data that validate lower radiation doses[28].

## PURE SEMINOMAS

### Stage I

In the current era, it is highly unusual for anyone with clinical stage I seminoma to die from the disease; 80% or more of patients are cured with inguinal orchiectomy alone and adjuvant, external-beam radiation administered either in a dog-leg or para-aortic field raises the relapse-free survival to at least 95%. Five-year disease-specific survival is about 99.5% and late relapses are rare. Men who forego radiation following orchiectomy and opt instead for a surveillance protocol have an 18–20% risk of relapsing, but they can almost always be cured with either radiation therapy or chemotherapy at the time of relapse and, thus, their 5-year disease-specific survival is also about 99.5%. With such high survival rates, short- and long-term toxicity from treatment gained attention from treating physicians and concerns developed with regard to the potential for radiation therapy to cause secondary malignancies decades after it was delivered. Because testicular seminoma patients are mostly between the ages of 30 and 50 years, most live long enough to be at risk for such late effects of treatment. Moreover, because only 20% of stage I seminoma patients are destined to relapse following orchiectomy, 80% of those treated with radiation are treated unnecessarily. With such a small fraction benefiting from radiation, treatment toxicity is difficult to tolerate.

Concerns about treatment toxicity fueled interest in surveillance and chemotherapy as alternatives to radiation therapy for stage I seminomas. Other strategies have been to reduce the radiation field size and dose and to develop risk stratification systems so that only those patients at highest risk for relapse receive treatment (while low-risk patients undergo surveillance). One strategy that is not typically offered to clinical stage I seminoma patients is RPLND. In theory, RPLND should be an excellent treatment option for seminomas because these tumors are particularly likely to spread lymphatically (i.e., to the retroperitoneum) rather than hematogenously; one would expect on that basis for post-RPLND relapses to be even less common for seminomas than nonseminomas. However, RPLND in seminoma patients has been associated with a higher rate of complications than in nonseminomatous tumors and with higher relapse rates than seen with radiation. The difficulty resecting seminomas appears to derive from the greater tendency of these tumors to exhibit extranodal extension and to provoke a scirrhous reaction with the result that removing the nodes is more challenging and the risk of leaving cancer behind is greater. However, the data on RPLND in early stage seminomas are scant and it is possible this question will be readdressed in the future.

### *Radiation Therapy and the Risk of Secondary Malignancies and Other Late Complications*

The risk of major late complications from radiation therapy for seminomas has been well documented. Adequately powered studies with sufficient follow-up have consistently reported an association between radiation therapy for GCTs and an increased incidence of solid malignancies, with the reported relative risk ranging from 1.45–7.5[29,30,31,32]. M.D. Anderson reported that men with seminomas treated with radiation had a relative risk of death from secondary malignancies of 1.91 (95% CI = 1.30–2.71). The largest study was an international pooled analysis of population-based cancer registries including 28,843 men with over 3300 who had survived over 20 years[29]. Among men receiving radiation for seminoma, the relative risk of second tumors was 1.45 overall and was 1.65 among those with 20-years of follow-up. Twenty-five years following treatment, 18% of seminoma patients had developed a secondary nongerm-cell cancer compared to an expected incidence of 9.3%. The implication is that for every 100 men treated with radiation for seminoma, there will be 9 excess nongerm-cell cancers. Putting this figure in context, if 100 men with clinical stage I seminoma are treated with radiation, only 18 to 20 stand to benefit because only 18 to 20 would relapse if put on a surveillance protocol. Of the 18 to 20 who would be expected to relapse, 5 will relapse despite receiving radiation, bringing the number who benefit down to 15. If 15 out of 100 treated are cured by treatment and 9 out of 100 develop radiation-induced malignancies that may be more difficult to cure than seminomas, the benefit-to-harm ratio of radiation is unfavorable. Current radiation therapy for stage I seminoma uses smaller fields and lower treatment doses than were used previously. Whether these changes will result in substantially fewer radiation-induced cancers remains to be seen.

No increased risk of cancer has been reported in men with seminomas treated with chemotherapy. An analysis of 2006 testicular cancer survivors in Norway with a mean follow-up of 12 years also reported that radiation (RR = 1.58; 95% CI 1.3–1.9), but not chemotherapy (OR = 1.32, 95% CI 0.4–3.4), was associated with an increased risk of secondary malignancies[33]. However, the highest risk was seen among patients who had received both chemotherapy and radiation (RR = 3.54; 95% CI 2.0–5.8), an interaction that has been confirmed by other studies[34,35]. A Dutch report of 1909 testicular cancer survivors similarly reported that radiation therapy was associated with secondary cancers (RR = 1.8; 95% CI 1.4–2.2), but chemotherapy was not (RR = 0.83; 95% CI 0.10–3.0)[35]. Malignancies associated with radiation therapy include leukemia, sarcomas, and cancers of the stomach, bladder, colon, and pancreas. In contrast to other secondary malignancies, leukemia is more strongly associated with chemotherapy. Travis and colleagues reported that among 2664 patients receiving chemotherapy for nonseminomatous GCTs, 6 cases of leukemia were seen, representing a relative risk of 11 (95% CI = 4.18–25)[29]. However, because the baseline leukemia risk is so low, the elevated relative risk translates into fewer than 5 cases for every thousand patients treated with a standard regimen of 3 to 4 cycles of cisplatin-based chemotherapy. The incidence of leukemia following one or two cycles of adjuvant chemotherapy is unknown. The risk of leukemia in patients treated with modern GCT chemotherapy regimens appears to be related primarily to etoposide, but cisplatin probably contributes as well[36,37]. Carboplatin has not been associated with leukemia or other secondary cancers at the doses used for stage I seminoma.

Cardiovascular morbidity and mortality has also been associated with radiation therapy for testicular GCTs. A British study reported that among 992 men followed for a median of 10.2 years, 9.6% of those receiving radiation therapy and 6.7% of those receiving chemotherapy experienced a cardiac event, compared to only 3.7% of those treated by orchiectomy alone[38]. After adjusting for age, the relative risk was 2.59 for chemotherapy, 2.40 for radiotherapy, and 2.78 among those who received both. No difference in risk was seen when comparing cisplatin to carboplatin chemotherapy. Increased cardiac mortality per se has not been demonstrated in patients treated with chemotherapy, but a report from the M.D. Anderson Cancer Center on 447 men treated between 1951 and 1999 found that radiation therapy for seminoma was associated with a 61% increase in cardiac-specific mortality compared to the general population[32]. Whether modern radiation therapy, which uses lower doses and small fields, is associated with a similar risk remains unclear. However, the M.D. Anderson study reported that the increased risk of cardiovascular mortality was not limited to those receiving mediastinal radiation.

Radiation for seminoma has also been associated with peptic ulcer disease and infertility. Reported rates of peptic ulcers have ranged from 0–16%[4,39,40,41,42,43,44]. The MRC TE10 phase III study comparing para-aortic to dog-leg radiation fields collected toxicity data systematically and reported that, with a median follow-up of 4.5 years, 33 of 478 men (7%) were diagnosed with a peptic ulcer[42]. Regarding fertility, Huyghe and colleagues reported on a series of 451 men with GCTs who had either undergone orchiectomy followed by surveillance, RPLND, chemotherapy, or radiation[45]. Patients who had received radiation therapy had an odds ratio for achieving conception of 0.35 compared to men who had received chemotherapy ($p$ = 0.017) despite the use of testicular shielding. On the other hand, modern studies of stage I seminoma have reported that 60–90% of men who try to father children following radiation therapy are successful[45,46].

## Modern Radiation Therapy: Smaller Field Sizes and Lower Doses

Historically, the radiation field extended to include the mediastinum, supraclavicular region, para-aortic region, and ipselateral hemipelvis, but radiation above the diaphragm is now considered inappropriate with the risks of additional toxicity outweighing any small benefit in disease control. Multiple retrospective studies have reported a twofold or greater excess cardiac mortality among patients receiving mediastinal radiation[47,48,49] and it does not appear that mediastinal radiation significantly improves cancer control outcomes[50]. Prophylactic mediastinal radiation is, therefore, currently contraindicated.

Many centers omit treatment of the pelvis, using a para-aortic field only that typically extends from either the superior or the inferior border of the tenth vertebral body down to the L4-L5 or L5-S1 disk space[42,51]. The other commonly used field is the “dog-leg” or “hockey-stick” field, which uses the same superior margins as the para-aortic field, but extends more inferolaterally to the ipselateral pelvis. A randomized controlled trial conducted by the Medical Research Council assigned 478 men with clinical stage I seminoma to either para-aortic or dog-leg irradiation with a dose of 30 Gy[42]. With a median follow-up of 4.5 years, nine patients had relapsed in each arm, yielding a 3-year, relapse-free survival of 96.0% in the para-aortic field arm and 96.6% in the dog-leg field arm. Nausea, vomiting, diarrhea, and leukopenia occurred less often in the para-aortic arm and recovery of sperm counts was delayed in the dog-leg arm. However, four relapsing patients in the para-aortic arm relapsed in the pelvis while there were no pelvic relapses in the dog-leg arm. Use of a para-aortic field was thus associated with equivalent efficacy and less toxicity and was advanced as a new standard of care, but some institutions still use a dog-leg field due to concern about pelvic relapses. These excellent results with a para-aortic field have been confirmed in a large, prospective, single-arm, German study of 625 patients that reported a relapse rate of 5% and 8-year disease-specific survival of 99.6%[52]. Of the 21 men who relapsed, 10 had recurrent disease in the pelvis, a substantially higher figure than would have been expected with a dog-leg field. Whether it is appropriate to expose 625 men to pelvic irradiation in order to prevent 10 pelvic relapses is debatable although most centers have adopted the para-aortic field because the overall relapse rate and survival rate are identical with para-aortic and dog-leg fields.

The radiation dose used to treat seminomas has declined over time. While 30 Gy or higher was often used for clinical stage I disease in older trials, recent studies have reported equally favorable results with lower doses and many consider 20 or 25 Gy to be the standard dose today[53]. The Medical Research Council study TE18 randomized 625 patients to either 20 Gy in 10 fractions or 30 Gy in 15 fractions. With 61 months median follow-up, the relapse-free survival was 97% in the 30-Gy arm and 96.4% in the 20-Gy arm, a nonsignificant difference[28]. All patients were disease free or dead of unrelated causes except for one patient with widely metastatic disease in the 30-Gy arm and one patient who died of seminoma in the 20-Gy arm. A second randomized Medical Research Council trial, including 469 patients who were randomized to 30 or 20 Gy, reported 2-year, relapse-free survival of 96.8% with 30 Gy and 97.5% with 20 Gy[28]. Taken together, these studies exclude with 95% confidence an increase of greater than 0.5% in the relapse rate when dropping the radiation dose to 20 Gy[28]. A separate multicenter study of 431 men in the U.K. reported that treatment with 20 Gy resulted in a 5-year, relapse-free survival rate of 96.3% and a disease-specific survival of 99.8%, figures entirely comparable to studies of higher doses[54]. The TE18 study evaluated quality of life and reported a substantial improvement in acute side effects with the lower radiation dose. The rate of severe lethargy (20 vs. 5%) and the inability to carry out normal work activities (46 vs. 28%) was significantly higher in the 30-Gy arm ($p < 0.001$).

These studies would appear to support strongly the adoption of 20 Gy as the new standard radiation dose for stage I seminomas. Whether this dose reduction results in fewer secondary cancers will probably not be clear until long-term, follow-up data are available.

## Surveillance

Postorchiectomy surveillance is considered a standard option for men with clinical stage I pure seminomas. There is no evidence that opting for surveillance rather than immediate radiation or chemotherapy compromises long-term survival. Almost all relapsing patients are salvaged and 5-year, disease-specific survival ranges from 99–100%[4,5,9,55]. In an international pooled analysis of 638 patients, 5-year overall and disease-specific survival were 97.7 and 99.3%, respectively[56]. Surveillance offers patients the obvious advantage of avoiding postorchiectomy therapy unless it turns out to be necessary as a result of relapsed disease. Given the increased late mortality associated with radiation therapy due to excess cardiac events and secondary cancers, as well as the possibility of long-term side effects from carboplatin chemotherapy, the opportunity to avoid treating at least 80 out of 100 men is attractive. Moreover, low-risk subgroups can be identified who have only a 5–10% incidence of relapse, and these patients are particularly attractive candidates for surveillance[56,57]. Ninety percent of relapses are limited to the retroperitoneal lymph nodes and 75% of relapsing patients are managed with radiation therapy at the time of relapse. Fewer than 5% of patients on surveillance end up needing chemotherapy either at the time of first relapse or subsequently in a salvage setting. Thus, patients undergoing surveillance are no more likely to receive chemotherapy than stage I patients managed with radiation therapy. The main disadvantage of surveillance is that patient or physician noncompliance with the surveillance schedule can allow relapses to progress to a very advanced and less-curable stage before they are detected.

## Single-Agent Carboplatin Chemotherapy

The newest established treatment option for stage I seminomas is carboplatin chemotherapy[26]. In multiple phase II trials and one phase III trial involving over 1000 patients, disease-specific survival is 100% and relapse-free survival has been reported at less than 2% with two cycles of carboplatin compared to about 5% when only one cycle is given[7,8,9,10,11,12,13,14,15,16]. Carboplatin in this setting has not been associated with grade 4 toxicity except for a rare incidence of thrombocytopenia.

Results from seven phase II trials of *two cycles* of single-agent carboplatin are available, including data from 650 men[7,8,9,10,14,15,57,59]. Only 11 (1.7%) have relapsed and all of these have been successfully salvaged with additional therapy. None has died of seminoma or acute treatment toxicity; disease specific survival is 100%. These low relapse rates have been particularly impressive because some trials have restricted carboplatin to high-risk patients with the greatest risk of relapse if put on a surveillance protocol[9,57]. Looking only at studies with a mean follow-up of at least 5 years, only 3 of 272 men (1.1%) have relapsed and all have relapsed within 2 years of receiving carboplatin[10,14,15]. While a risk of late relapses has been posited as a theoretical concern, no late relapses have been documented thus far. The European Oncology Research and Treatment Group and Medical Research Council randomized phase III trial comparing radiation to a single dose of carboplatin dosed at an AUC of 7 reported its preliminary results in abstract form in 2004[13]. With 1447 men enrolled and a median follow-up of 3 years, relapse-free survival was 96.6% in the radiation arm and 95.4% in the carboplatin arm and the 95% confidence intervals excluded an increased risk in the carboplatin arm of more than 4%. No disease or treatment-related deaths were reported in the carboplatin arm and only one was reported in the radiation arm. Seven patients receiving radiation developed second germ-cell cancers compared to only one patient receiving carboplatin. This trial appears to establish equivalency between *a single cycle* of carboplatin and radiation for stage I seminoma, but mature results are needed. This is the second study, however, to suggest that a single cycle of carboplatin may result in a higher relapse rate than two cycles[11].

Carboplatin also looks favorable from a toxicity perspective. No cases of febrile neutropenia have been reported and carboplatin has never been associated with secondary malignancies. Two patients in the published seminoma series developed severe thrombocytopenia and received platelet transfusions, but no bleeding complications have been reported. In a randomized phase II trial, Oliver reported that chemotherapy resulted in greater myelosuppression and altered taste than radiation, but that radiation resulted in greater nausea, diarrhea, and fatigue[10]. Assessing the impact of testicular cancer chemotherapy on fertility represents a challenge because most men have abnormal sperm counts at the time of diagnosis and testicular cancer is associated with infertility both prior to diagnosis and following treatment with orchiectomy alone[60,61,62,63,64]. After treatment, sperm counts typically return toward normal. Reassuring data were published by Reiter et al., who found that normospermia increased from 35% following orchiectomy to 68% following chemotherapy[65]. Similarly, no renal or neurological toxicity has been documented. Multiagent, platinum-based chemotherapy for testicular cancer has been associated with dyslipidemia, hypertension, and an increased, long-term risk of cardiovascular events in some[31,38,66,67,68]. It has not been demonstrated that patients receiving chemotherapy have a higher cardiovascular mortality than the general population. The vast majority of patients in these studies received cisplatin, rather than carboplatin, but one study that evaluated both agents found no evidence that carboplatin was safer in the context of a multidrug regimen[38]. There are inadequate long-term follow-up data to assess whether two cycles of single-agent carboplatin have any impact on vascular health, but this is an issue that clearly warrants future monitoring.

## *Conclusion*

In 2005, there are three standard treatment options for men with clinical stage I pure seminomas of the testis: surveillance, external-beam radiation therapy, and carboplatin chemotherapy. Each is associated with perfect or near-perfect, 5-year, disease-specific survival. Radiation therapy has been associated with secondary malignancies, cardiovascular mortality, and peptic ulcer disease, but has a long track record as a highly effective treatment for seminoma. Surveillance allows 80% of men to avoid any postorchiectomy therapy, but if a man does relapse on surveillance, the treatment administered is more aggressive than the treatment administered for stage I disease. Single-agent carboplatin chemotherapy offers the lowest published relapse rates (if two cycles are given) and has not been associated with secondary malignancies or other life-threatening complications. However, carboplatin is a newer treatment for seminoma and there are fewer patients with very long-term follow-up than there are for radiation or surveillance; whether there are any late complications manifesting at 15–20 years following treatment with carboplatin remains unknown.

## Stage II

No major changes have occurred in the past decade in the management of stage II seminomas. For early stage IIA disease, standard treatment is external-beam radiation therapy while for stage IIC disease, multiagent cisplatin-based chemotherapy (three cycles of BEP or four cycles of etoposide plus cisplatin) is preferred. The cure rate with radiation declines as tumor bulk increases. Radiation therapy has less acute toxicity than cisplatin-based chemotherapy and is thus generally preferred when it can achieve equivalent cure rates, as it does for nonbulky disease. Chemotherapy is preferred for bulky disease because it produces lower relapse rates. The specific disease bulk criteria at which to choose chemotherapy over radiation has not been clearly established. Moreover, how the immediate risk of neutropenic sepsis and other acute chemotherapy toxicity balances against the risk of radiation-induced secondary malignancies is unclear.

The absence of any randomized controlled trials makes it difficult to establish a definitive recommendation. Smalley et al. reviewed their radiation experience with stage II seminomas and reported that among 340 men treated for nonbulky disease, 30 (9%) relapsed and 322 (95%) survived their disease[69]. In contrast, among 356 men treated for bulky stage II disease, 124 (35%) relapsed. The Princess Margaret Hospital has recently reported similar results, with 5-year, disease-free survival rates of

92% for stage IIA and 90% for stage IIB disease following radiation[70]. However, 9 of 16 (56%) stage IIC patients treated with radiation subsequently relapsed compared to only one of 23 (4.3%) IIC patients treated with chemotherapy ($p = 0.0004$).

Modern radiation therapy for stage II disease uses a dog-leg field as described for stage I seminoma. Historically, an inverted Y field was previously often used such that the pelvis was treated bilaterally, but contralateral pelvic relapses are uncommon and this is no longer considered necessary. Similarly, as discussed for stage I disease, supradiaphragmatic radiation has been abandoned by most centers. The radiation dose is similar to that used for stage I disease with the enlarged lymph nodes receiving a boost to a total dose of 35 Gy. Chemotherapy for bulky disease is identical to treatment of favorable risk disseminated disease: three cycles of BEP or four cycles of etoposide and cisplatin[58,71,72,73]. Five-year overall and relapse-free survival in stage IIC patients following chemotherapy has been reported at 85 and 78%, respectively, or higher[70,73].

Patients with stage IIA or IIB seminomas who have contraindications for radiation therapy should receive chemotherapy for advanced stage disease. These include patients with either a horseshoe or pelvic kidney or patients with disease sufficiently lateral in location to necessitate significant radiation exposure by one of the kidneys or the liver[74].

## NONSEMINOMATOUS GERM CELL TUMORS

A GCT with any component of yolk-sac tumor, embryonal carcinoma, teratoma, choriocarcinoma, and/or undifferentiated GCT is considered in the category of nonseminomatous germ cell tumors (NSGCTs) and most NSGCTs contain a mixture of different GCT subtypes, often including a component of seminoma. Chemotherapy is playing a growing role in the management of early stage NSGCTs, just as it is in early stage seminomas.

### Clinical Stage I

Over the past 5 years, chemotherapy as primary postorchiectomy therapy for clinical stage I NSGCTs has gained acceptance as a standard treatment option in addition to the alternatives of surveillance and RPLND[26,27]. Each of these three options is associated with a 5-year, overall survival of about 99% (Table 2). In Europe, there is distinctly less enthusiasm for RPLND than there is in the U.S.[26]. However, recent data suggest modern RPLND results are even better than the excellent historical results[2]. As with seminomas, the case for surveillance is that it allows most men to avoid any postorchiectomy treatment without any sacrifice with regard to long-term survival. The case for chemotherapy has been that it results in the lowest relapse rates, while RPLND has the advantage of sparing men the toxicity of chemotherapy and minimizing the risk of late relapse. There is no meaningful evidence than any of these treatment options results in superior long-term outcomes than the others (Table 2).

#### *Surveillance, RPLND, and Adjuvant (Primary) Chemotherapy*

The following results are seen for the three treatment options.

##### Surveillance

About 30% of men on surveillance will relapse, but most can be cured with treatment at relapse such that the 5-year, disease-specific survival rate is 99%. Most men have elevated serum tumor markers with or without retroperitoneal adenopathy as their manifestation of relapse and receive cisplatin-based chemotherapy for disseminated disease[75,76,77,78,79,80,81,82] (Table 2).

## RPLND

The relapse rate for men undergoing RPLND differs according to their pathological stage and their serum tumor marker levels prior to surgery. Disease specific survival at 4–5 years exceeds 99% at centers of excellence[2,79,83,84,85]. The main complication is anejaculation seen in about 5% of men. Major surgical complications are rare.

Historically, men with stage I disease and elevated serum tumor markers were considered eligible for RPLND, but such men suffered high relapse rates and elevated serum HCG or AFP is currently seen as a contraindication for RPLND for stage I disease[1,2,86]. Instead, patients with persistently elevated markers are now treated as having disseminated disease and receive three to four cycles of cisplatin-based chemotherapy. Among clinical stage I patients undergoing RPLND, 70–77% are found to be pathological stage I, while lymph node metastases are found in the remainder. Most men with pathological stage II disease end up receiving adjuvant chemotherapy[2]. These figures are remarkably consistent with the 25–30% relapse rate seen in men on surveillance. Among men with normal serum tumor markers and pathological stage I disease, a relapse of 3% was reported in the most recent large series[2]. Older studies reported a relapse rate of about 10% in this population[2,83,85]. Men with clinical stage I, pathological stage IIA disease, and normal pre-RPLND serum tumor markers have a relapse rate of 10–20%[2,87]. Thus, in a hypothetical sample of average risk clinical stage I patients undergoing retroperitoneal lymph node dissection, at least 70 men will be treated unnecessarily because they were cured with orchiectomy alone. Of the remaining 30, about 19 or 20 will end up receiving chemotherapy, either due to the detection of lymph node metastases at RPLND or due to subsequent relapse[2]. Thus, primary RPLND results in about a 10% absolute reduction and a 33% relative reduction in the risk of needing chemotherapy compared to surveillance in the average patient (Table 2).

Men with pathological stage IIB-C disease have a relapse rate of 50–70%[2,88] and these men are, therefore, generally advised to undergo adjuvant chemotherapy following RPLND because relapse-free survival following adjuvant chemotherapy is over 98%[88,89,90]. As discussed below, men with a predominance of embryonal carcinoma appear to have a higher risk of relapse following RPLND, particularly if lymphovascular invasion is present in the primary tumor. Memorial Sloan-Kettering reported that 73% of clinical stage I patients with pure embryonal carcinoma undergoing RPLND had lymph node metastases[91]. Most concerning, 53% of clinical stage I patients had relatively bulky (pN2 or pN3) disease. As a result, 55% of the patients in the series received adjuvant chemotherapy for pathological stage II disease and another 2% received chemotherapy for relapse. Most patients with pure or predominant embryonal carcinoma thus end up receiving chemotherapy despite RPLND.

## Adjuvant (Primary) Chemotherapy

Chemotherapy following orchiectomy for clinical stage I disease results in the lowest relapse rates in case series and phase II trials. Nine studies including a total of 579 men reported that only 16 (2.8%) relapsed and disease-specific survival was 99%[17,18,19,20,21,22,23,24,25] (Table 2). Adjuvant chemotherapy for stage I disease typically consists of two cycles of BEP. Toxicity includes an average decline in pulmonary diffusion capacity of 15% and a 12% incidence of high-pitch hearing loss[17]. This amount of chemotherapy does not appear to have any significant impact on sperm count or motility. Potential late toxicity is not well documented for two cycles of BEP, but BEP in general has been associated with an increased risk of acute myelogenous leukemia and cardiovascular disease. The risk of leukemia for men receiving up to four cycles of BEP is estimated at between 2 and 4 cases per 1000 men treated[37,94]. Men receiving BEP or other cisplatin-based chemotherapy have been reported to have a risk for cardiac events of 2.6–7 times higher than controls, but most patients in these series received at least 3 or 4 cycles of chemotherapy and the risk of two cycles of BEP is not known.

## *Risk Stratification*

On average, the risk of relapse following orchiectomy for a clinical stage I testis cancer with normal postorchiectomy serum tumor markers (AFP, LDH, and beta-hCG) is about 30%. However, subgroups can be identified with lower and higher risks. Predictors of relapse include the presence of lymphovascular invasion, a high proportion of embryonal carcinoma, and the absence of yolk sac tumor. Pure teratomas have a lower relapse rate than average. The Medical Research Council (MRC) identified and then prospectively validated four independent risk factors for relapse: vascular invasion, lymphatic invasion, the presence of embryonal carcinoma elements, and the absence of yolk sac tumor[75,76]. However, even among men with three or four of the risk factors, fewer than half (47%) relapsed. With regard to identifying low-risk patients, 24 of 141 (17%) men with 0–1 risk factors relapsed and these low-risk men represented 39% of the study participants. Of men with two risk factors, 21% relapsed and this group represented 39% of the participants, which is similar to the average relapse rate of stage I nonseminoma patients in general. The MRC approach was, thus, of limited utility in identifying appropriate patients for surveillance and for treatment: over half those identified as high risk would be treated unnecessarily while four out of ten men could not be classified as either high or low risk.

Subsequent efforts at risk classification have identified lymphovascular invasion (LVI) and embryonal carcinoma (EC) as the two elements most predictive of relapse[77,78,80,81,85,91, 93,94,95,96]. However, prospective validation of models other than those of the MRC are lacking with one exception discussed below[22]. Taking into account the proportion of the tumor that is EC rather than simply the presence or absence of this tissue type has led to models with stronger predictive power[94]. In a retrospective study of men with clinical stage I disease undergoing RPLND, Moul and colleagues reported that a model combining the percent EC and the presence or absence of LVI could accurately categorize 88% of the men as pathological stage I or II (i.e., having or lacking lymph node metastases)[93]. They reported that occult retroperitoneal lymph node metastases can be demonstrated in 89% of clinical stage I patients whose tumor showed either more that 80% EC or the combination of LVI and more than 45% EC. Similarly, Indiana University reported that 62% of men whose tumors showed LVI and a predominance of EC were found to have metastatic disease subsequently[96], while Memorial Sloan-Kettering Cancer Center reported that 73% of men with pure EC had metastases detected at RPLND[91]. Attempts have also been made to utilize PET scans and other imaging modalities to predict which stage I patients are likely to relapse, but the results have been disappointing thus far and there is no validated use of PET scans for stage I patients at this time.

Men with a low risk of occult metastatic disease have also been identified[97]. In the Indiana series, 84% of men with clinical stage I disease and neither LVI nor a predominance of EC remained without evidence of metastatic disease with at least 2 years of follow-up[96]. In Moul's model, men with either less than 45% EC or the combination of no LVI and less than 80% EC had only a 13% risk of having metastases detected at RPLND[93]. The presence of mature teratoma has also been associated with a low risk of occult metastases[82], but in multivariate analyses, mature teratoma has generally been eclipsed by LVI and the proportion of EC. One exception to this was a prospective trial that put low-risk men on surveillance. Patients were considered low risk if they had (1) no lymphovascular invasion by the tumor, (2) less than 80% EC, and (3) at least one of the following two criteria: a preorchiectomy serum AFP less than 80 ng/dL or more than 50% teratoma in the primary tumor[22]. In this study, only 2 of 23 low-risk patients relapsed (8.7%). A different series, however, showed that men with clinical stage I pure mature teratomas of the testicle have a significant risk of metastatic disease; one representative series reported that 5 of 26 patients (19%) had lymph node metastases discovered at RPLND[98]. It is not at all clear, however, that all men with teratoma discovered at RPLND would have relapsed without undergoing surgery. While teratoma is generally less chemosensitive than other GCT elements, the relapse rate following postorchiectomy chemotherapy is far lower than would have been predicted from the number of men found to have teratoma in lymph nodes at RPLND. Specifically, 21% of men are found to have teratoma at RPLND for stage I NSGCTs, but fewer than 3% of men relapse following adjuvant chemotherapy[2](Table 2). Therefore, when estimating relapse rates for men undergoing surveillance, it is probably more reliable to use data from surveillance series showing actual relapse rates rather than trying to predict relapse rates based on pathological findings from men undergoing RPLND instead of surveillance. Unfortunately, much of the recent prognostic literature has been based on RPLND series rather than surveillance series.

Risk stratification can be used to identify subgroups who appear to be better candidates for one of the three treatment options[22,82,84]. The benefit of surveillance is greatest for men who have a low risk of relapse given that relapse during surveillance usually results in more aggressive treatment than would be given for stage I disease following orchiectomy. In contrast, the rationale for adjuvant chemotherapy is clearest in men with a high risk of relapse in that the early and late toxicity of chemotherapy is easier to accept in patients who have a high probability of relapsing without treatment. RPLND may seem an unattractive option for men with a predominance of EC and LVI because most of them end up receiving chemotherapy after RPLND, so it may make more sense to give them the chemotherapy up front and spare them a major abdominal operation. Indiana University reported that a 30% post-RPLND relapse rate is seen in these men even when no cancer is found in the resected lymph nodes (i.e., pathological stage I), and Memorial Sloan-Kettering reported that over half of all clinical stage I patients with pure EC undergoing RPLND end up receiving subsequent chemotherapy. Similarly, M.D. Anderson reported that 42% of high-risk clinical stage I patients relapsed following RPLND, including 37% of those who were pathological stage I[22]. In other words, RPLND in these patients does not succeed in allowing them to avoid chemotherapy, while adjuvant chemotherapy results in a relapse rate of less than 3% even in high-risk patients[17,23]. However, there is no consensus regarding treatment algorithms for these patients.

### Stage II Nonseminomatous Germ Cell Tumors

There have been no major recent changes in the management of stage II tumors. In general, stage IIA NSGCTs with normal serum tumor markers are managed with RPLND, while stage IIB and IIC are managed as disseminated testicular cancer with cisplatin-based chemotherapy. Any patient with elevated postorchiectomy serum tumor markers is typically treated as having disseminated disease. The recent report from Memorial Sloan-Kettering Cancer Center illustrates the logic behind this approach: among patients who did not receive any chemotherapy, only 10% of clinical stage pN1 patients relapsed following RPLND whereas 70% of pN2 patients suffered a relapse[2]. However, the treatment of clinical stage IIB and IIC patients typically requires both chemotherapy and RPLND, and thus the question is which order is preferable: chemotherapy followed by resection of residual disease or resection followed by adjuvant chemotherapy. One of the main issues driving the preference for providing chemotherapy first is the concern that systemic micrometastases could progress while waiting for the patient to undergo and recover from surgery. There are no randomized trials comparing these two approaches and the specific criteria by which to decide whether a patient is better served by RPLND or chemotherapy are not well defined.

## CONCLUSION

Early stage testicular GCTs have an outstanding prognosis and deaths from this disease have been rare. However, as the studies discussed above demonstrate, treatment-related toxicity has become a greater concern with long-term follow-up, particularly with regard to radiation-induced malignancies in stage I seminoma patients receiving radiation therapy. Single-agent carboplatin appears to offer an attractive alternative to radiation therapy, but it remains unknown whether carboplatin is associated with late toxicity. For nonseminomas, the debate continues about the relative merits of RPLND vs. chemotherapy, but it is clear that a substantial number of stage I men who undergo RPLND end up receiving chemotherapy, too. Nonetheless, there are concerns about the potential for late toxicity at the doses used in this setting[100] from chemotherapy and RPLND results in the fewest possible number of men receiving chemotherapy. The concern that men undergoing adjuvant chemotherapy rather than RPLND would end up suffering late relapses due to unresected metastatic teratoma has not been validated by the published studies, but there are limited long-term follow-up data is available. One major goal for the future is to improve our prognostic capabilities with regard to which men are cured by orchiectomy alone; it would then be possible to limit treatment, and the toxicity thereof, to those destined to relapse.
